# Divalent Cations and Redox Conditions Regulate the Molecular Structure and Function of Visinin-Like Protein-1

**DOI:** 10.1371/journal.pone.0026793

**Published:** 2011-11-02

**Authors:** Conan K. Wang, Anne Simon, Christian M. Jessen, Cristiano L. P. Oliveira, Lynsey Mack, Karl-Heinz Braunewell, James B. Ames, Jan Skov Pedersen, Andreas Hofmann

**Affiliations:** 1 Structural Chemistry Program, Eskitis Institute for Cell & Molecular Therapies, Griffith University, Brisbane, Queensland, Australia; 2 Universite Lyon 1, Institut de Chimie et Biochimie Moleculaires et Supramoleculaires, Villeurbanne, France; 3 Complex Fluids Group, Department of Experimental Physics, University of São Paulo, São Paulo, Brazil; 4 Department of Chemistry and iNANO Interdisciplinary Nanoscience Centre, University of Aarhus, Aarhus, Denmark; 5 School of Biological Sciences, The University of Edinburgh, Edinburgh, Scotland, United Kingdom; 6 Molecular & Cellular Neuroscience Laboratory, Biochemistry & Molecular Biology Department, Southern Research Institute, Birmingham, Alabama, United States of America; 7 Department of Chemistry, University of California Davis, Davis, California, United States of America; University of Queensland, Australia

## Abstract

The NCS protein Visinin-like Protein 1 (VILIP-1) transduces calcium signals in the brain and serves as an effector of the non-retinal receptor guanylyl cyclases (GCs) GC-A and GC-B, and nicotinic acetyl choline receptors (nAchR). Analysis of the quaternary structure of VILIP-1 in solution reveals the existence of monomeric and dimeric species, the relative contents of which are affected but not exclusively regulated by divalent metal ions and Redox conditions. Using small-angle X-ray scattering, we have investigated the low resolution structure of the calcium-bound VILIP-1 dimer under reducing conditions. Scattering profiles for samples with high monomeric and dimeric contents have been obtained. The dimerization interface involves residues from EF-hand regions EF3 and EF4.

Using monolayer adsorption experiments, we show that myristoylated and unmyristoylated VILIP-1 can bind lipid membranes. The presence of calcium only marginally improves binding of the protein to the monolayer, suggesting that charged residues at the protein surface may play a role in the binding process.

In the presence of calcium, VILIP-1 undergoes a conformational re-arrangement, exposing previously hidden surfaces for interaction with protein partners. We hypothesise a working model where dimeric VILIP-1 interacts with the membrane where it binds membrane-bound receptors in a calcium-dependent manner.

## Introduction

Neuronal calcium sensor (NCS) proteins play key roles in controlling neuronal function [1], and have been implicated physiologically in synaptic plasticity [2], [3], neuropathological processes [4], [5], pain modulation [6], and cancer [7]. The NCS protein Visinin-like Protein 1 (VILIP-1) has been hypothesised to affect neuronal signalling in a calcium and cyclic guanosine monophosphate (cGMP)-dependent way [8]. Effects of VILIP-1 on the non-retinal receptor guanylyl cyclases (GCs) GC-A and GC-B, as well as soluble GCs have been observed *in vitro* [9].

It is believed that cGMP synthesis by guanylyl cyclases requires dimerisation, since a functional catalytic site is only obtained by association of two polypeptide chains within the dimer [10]. Co-localisation of VILIP-1 with GC-B in hippocampal neurons has been observed [11], and the protein has also been shown to interact directly with the catalytic domains of GC-A and GC-B using GST pull-down assays and surface plasmon resonance [9]. We thus follow the hypothesis that VILIP-1 dimerisation is of functional importance for its biological activity. This notion receives further support by observations with other NCS proteins where reversible dimerisation is an essential functional feature. Dimerisation of Guanylyl cyclase-activating protein-2 (GCAP-2) is required for activation of the photoreceptor membrane GC [12]. Also, the DNA binding of KChIP3/DREAM is regulated by Ca^2+^/Mg^2+^-mediated dimerisation of the protein [13].

**Figure 1:**
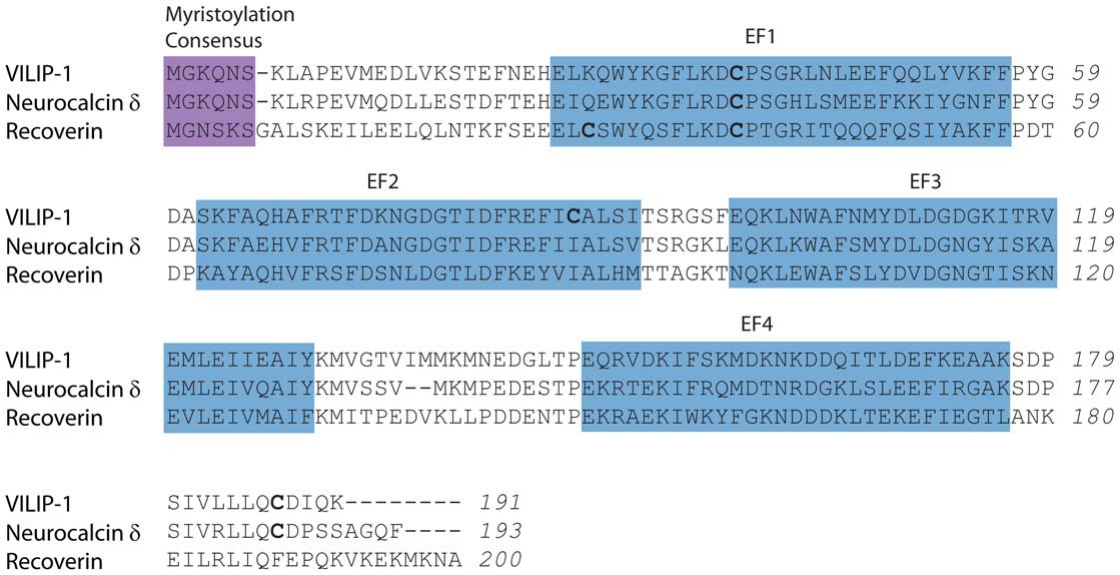
Amino acid sequence alignment of VILIP-1 and selected NCS proteins. The VILIP sub-family of NCS proteins include VILIP-1, -2, -3, neurocalcin δ and hippocalcin. The sequences of human VILIP-1, neurocalcin δ and recoverin are shown in the alignment. All proteins shown in the alignment contain an amino-terminal myristoylation consensus sequence, which is highlighted in purple. They also have four EF-hand motifs (EF1-4), which are boxed and labeled. Cysteine residues are in bold. VILIP-1 contains three Cys residues.

Proteins of the VILIP subfamily of NCS proteins, including VILIP-1, -2, -3, neurocalcin δ and hippocalcin, share about 30–60% amino acid sequence identity with other NCS proteins, but between 67% and 94% among each other (for a review see [14]). VILIPs possess an M-G-X_3_-S consensus sequence for N-terminal myristoylation. The conjugated myristoyl group is subject to the ‘calcium-myristoyl switch’ which has been analysed in detail for recoverin where apo- and calcium-bound three-dimensional structures were first available [15]. As shown in Figure 1, VILIPs, like all NCS proteins, are constituted by four EF-hand motifs (EF1-EF4). EF1 is the most variable part in the sequence of NCS proteins, and is thus believed to be a possible interaction site with target proteins, with experimental support coming from the reported interaction of parts of EF1 and EF2 in GCAP-1 [16] and GCAP-2 [17] with retinal guanylyl cyclase. In most NCS proteins, including VILIP-1, the first EF-hand does not bind calcium. Interestingly, despite the presence of three remaining canonical EF-hands (EF2-EF4), VILIP-1 has been shown to bind only a total of two calcium ions per molecule [18].

Previous efforts have tried to identify residues of VILIP-1 that are important for dimerisation. A study focusing on the Redox-mediated dimerisation of VILIP-1 proposed that Cys187 is involved in a disulfide-linked VILIP-1 dimer [19]. In a more recent study, a model of the Redox-independent VILIP-1 dimer was proposed by computational docking, implicating residues between EF3 and EF4 in the dimerisation [20].

In the current study, we provide further insights into VILIP-1 dimer formation and highlight that VILIP-1 exists as a complex mixture of monomeric and dimeric species in solution with dimerisation being mediated by Redox-dependent and -independent mechanisms. Based on small-angle X-ray scattering (SAXS), we provide experimental evidence for the overall shape of dimeric calcium-bound VILIP-1 under reducing conditions, which is distinctly different from the dimer structures proposed for other NCS proteins, including recoverin [21], neurocalcin δ [22] and DREAM [23]. The VILIP-1 dimer structure obtained from solution scattering is in agreement with a model that was recently proposed based on NMR data [20]. We suggest that the dimer is important for the molecular function of VILIP-1 by facilitating its membrane association, and we characterized this by conducting monolayer adsorption experiments. Based on the low resolution structure obtained in this study and our membrane-binding results, we propose a molecular mechanism for VILIP-1 and its interaction with potential functional partners, e.g. GC.

## Materials and Methods

### Preparation of recombinant VILIP-1

The cDNA of VILIP-1 in pET8c [18] was transformed into competent *E. coli* BL21(DE3) cells. Expression of unmyristoylated VILIP-1 followed an in-house adaptation of the auto-induction protocol described by Studier [24]. A total of 8 L of LB auto-induction medium (0.1 mg L^−1^ ampicillin) were inoculated with an overnight culture of 1 L. The cells were grown at 37°C for 4 hours; incubation was then continued over night at 30°C. For production of myristoylated VILIP-1, BL21(DE3) cells were co-transformed with both pET8c-VILIP-1 and pBB131 vector encoding yeast N-myristoyltransferase (NMT). Cells were grown in a total of 2L of LB medium (0.1 mg L^−1^ ampicillin, 0.05 mg L^−1^ kanamycin) at 37°C until the optical density (A_600_) of the cell culture reached 0.6. Myristic acid was added to a final concentration of 0.2 mM and the culture was left to incubate for 0.5 hr. Expression was induced by adding isopropyl β-D-1-thiogalactophyranoside (IPTG) to the cell culture at a final concentration of 0.25 mM and the cells were grown at 25°C for 16 hr before harvesting.

After harvest, the cells were resuspended (100 mM NaCl, 1 mM EDTA, 20 mM TRIS (pH 8), 0.1% Triton X-100, 1 mM PMSF, 5 mM benzamidinium chloride), and lysed by multiple freeze-thaw cycles and subsequent sonication. The resulting suspension was cleared by ultracentrifugation (100000 g, 30 min, 4°C). The supernatant from the ultracentrifugation step was then dialysed against 20 mM TRIS (pH 8), and subjected to anion exchange chromatography using a QA52 column and a gradient of 0–1 M NaCl in 20 mM TRIS (pH 8). Appropriate fractions were pooled and dialysed against 100 mM NaCl, 1 mM MgCl_2_, 1 mM CaCl_2_, 0.1 mM dithiothreitol (DTT) and 20 mM HEPES (pH 7.5). The dialysed sample was then further purified by hydrophobic interaction chromatography using a phenyl sepharose column and isocratic elution with a buffer consisting of 100 mM NaCl, 2 mM ethylenediaminetetraacetic acid (EDTA), 0.1 mM DTT, 20 mM HEPES. After pooling appropriate fractions, the protein sample was concentrated and the buffer exchanged to 100 mM NaCl, 20 mM HEPES (pH 7.5). Protein quality was monitored throughout all purification procedures using denaturing SDS-PAGE.

### Mass spectrometry

The purified protein was identified by mass spectrometric fingerprinting using a Shimadzu Axima-LNR MALDI-TOF instrument. Protein samples of 1 mg/mL concentration were incubated for 22 hours with 0.02 mg/mL trypsin (Roche) in 25 mM ammonium bicarbonate 12 hours at 32°C. 0.5 µL of the digested sample were applied together with 0.5 µL of α-cyano-4-hydroxycinnamic acid onto the MALDI sample grid. The list of peptide masses obtained from the experiment was analysed using a program provided by the manufacturer, and peaks were compared to the Mascot database [25]. Final purified myristoylated VILIP-1 samples contained less than 5% of unmyristoylated protein as judged by mass spectrometry.

### Monolayer Adsoption

Measurement of protein adsorption to phospholipid monolayers was carried out using a computer-controlled Langmuir film balance (NIMA Model 301A) at 20°C. The area of the trough was 30×4 cm, and the instrument was equipped with a movable barrier that allowed adjustment of the surface area of the monolayer. The subphase buffer containing 2 mM CaCl_2_ or 2 mM EDTA, 100 mM NaCl, 20 mM HEPES (pH 7.5) was filtered (0.1 mm), and poured into the trough until the surface was 2 mm higher than the trough brim (∼100 mL). The surface of the buffer was separated by movable barrier into two isolated areas. A small Teflon stirrer, rotating at ∼40 rpm, was placed in the compartment where no phospholipids were added. The surface pressure was measured with a surface potential meter using 1×2.3 cm plates cut from filter paper (Whatman, No. 1). The lipid solution was prepared as a mixture of 1,2-dioleoyl-sn-glycero-3-phosphoserine (DOPS) and 1,2-dioleoyl-sn-glycero-3-phosphocholine (DOPC) (3∶1 molar ratio) dissolved in chloroform/methanol (2∶1 v/v) at a concentration of 1 mg mL^−1^, and ∼25 µL of this solution was applied onto the surface of the subphase with a Hamilton syringe. Surface pressure-area isotherms were acquired in separate experiments before each protein adsoption experiment to determine the best volume of lipid suspension required to construct the monolayer. After spreading, the monolayer was left to equilibrate for 20 min, and subsequently compressed by moving the barrier to generate a surface pressure π_0_ of ∼15–17 mN m^−1^. The protein was injected into the subphase at a final concentration of 30 nM using a Hamilton syringe extending beneath the barrier. The surface pressure π was recorded as a function of time for ∼45 min. Adsorption data were analysed with the software SDAR from the PCSB program collection [26].

### Size exclusion chromatography

Size exclusion chromatography was carried out using a Sephadex 200 column (GE Healthcare) on a BioLogic HPLC system (BioRad) with a standard protein buffer (100 mM NaCl, 20 mM HEPES, pH 7.5), previously calibrated with protein standards. All experiments were performed at a flow rate of 0.5 mL/min. Samples were incubated with varying additives (see Table 1) at least 2 hours before injection, and standard protein buffer containing the same additives was used for elution. After equilibrating the column, 1 ml of sample was loaded and elution of the protein was monitored by UV absorbance at 280 nm. The chromatograms were analysed using the program SDAR [26] to determine the position of and area under the elution peaks.

**Table 1:** Parameters derived from X-ray scattering for calcium-bound reduced VILIP-1.

ρ* [mg/mL]	12	6	3
*c* [µM]	542	271	136
*r_max_* [Å]	104	85	260
*R_g_* [Å]	30.2±0.2	25.9±0.2	63±5
*M* [kDa]	41.6±0.1	25.9±0.1	∼44

Buffer conditions: 100 mM NaCl, 5 mM CaCl_2_, 2.5 mM DTT, 20 mM HEPES (pH 7.5).

### Small-angle X-ray scattering (SAXS)

Measurements were performed on the instrument (modified NanoSTAR, Bruker-AXS) at the University of Aarhus [27]. Data collection was performed at 20°C in re-usable thermostated quartz capillaries, which are placed in the integrated vacuum chamber of the camera. Home-built capillary holders with good thermal contact to the thermostated surrounding block were used.

The sample-to-detector distance was 65 cm, which covered a momentum transfer range of 0.008<*q*<0.34 Å^−1^, where *q* = (4πsinθ)/λ2θ is the scattering angle, and λ is the radiation wavelength. The data sets were recorded using a two-dimensional position-sensitive gas detector (HiSTAR, Bruker-AXS). The measured data were corrected for variations in detector efficiency as well as spatial distortions, and were azimuthally averaged (Bruker-AXS SAXS software). Background scattering from 100 mM NaCl, 20 mM HEPES (pH 8.0) was subtracted and the scattering intensities were transformed to absolute units using the scattering of water as standard (SUPERSAXS package, Oliveira and Pedersen, unpublished). Data were recorded at ρ*, ρ*/2 and ρ*/4, with ρ* = 12 mg mL^−1^.

Measurement time of each concentration was 4 h. The radius of gyration *R_g_*, intensity of forward scattering *I*(0), and the distance distribution function *p(r)* were calculated using an Indirect Fourier Transformation procedure [28], [29], and *ab initio* models were obtained using GASBOR [30]. Theoretical scattering curves and their fit to experimental data were obtained with CRYSOL [31]. The experimental molecular mass was calculated from the intensity of forward scattering *I*(0) using the formula:
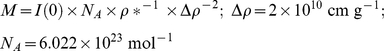
Δρ is the excess scattering length per unit mass of the protein and *N_A_* is Avogadro's number.

### Molecular modelling

The experimentally determined SAXS data was used to refine predicted models of the VILIP-1 monomer and dimer. For rigid body refinement of protein oligomers against SAXS data using a Monte-Carlo approach, we have generated the software SAFIR as part of the Java package PCSB [26]. The software in its current form applies random rotational and translational changes to individual components of a given oligomer to produce a new oligomer model. The new model is checked for steric clashes, and its agreement with the SAXS scattering data is evaluated using the χ value calculated by CRYSOL [31]. If accepted, the new model is subjected to a positional change in the next iteration. χ is defined as
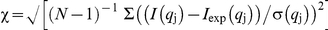
where *N* is the number of measured data points, *I*(*q*_j_) is the model intensity, *I*_exp_(*q*_j_) is the experimental intensity, and σ(*q*_j_) are the errors on the experimental intensities from counting statistics.

The generation of the homology model of the VILIP-1 monomer has been described previously [20], [32]. In short, the three-dimensional structure of neurocalcin δ (PDB accession number 1BJF) was used as a template for comparative modelling to generate the calcium-bound model, whereas the three-dimensional structure of recoverin (PDB accession number 1IKU) was used as a template to model the calcium-free structure. For both the calcium-bound and calcium-free models, twenty independent models were calculated with MODELLER [33], and the one with the lowest energy was selected, and its geometry scrutinised with PROCHECK [34]. Visual inspection and conformational adjustments were carried out with program O [35]. The model of calcium-bound VILIP-1 was then fitted into the SAXS scattering data using PCSB [26].

To model the calcium-bound VILIP-1 dimer, initial assemblies were generated using protein docking and experimentally-derived restraints [20], and fitted to the SAXS scattering data using manual and computational rigid body refinement as implemented in the in-house program SAFIR (see above). A calcium-free VILIP-1 dimer (see Discussion) was modeled by superimposing two individual calcium-free monomer models (as described above) on the selected model of calcium-bound VILIP-1.

## Results

### Membrane-binding Activity

**Figure 2:**
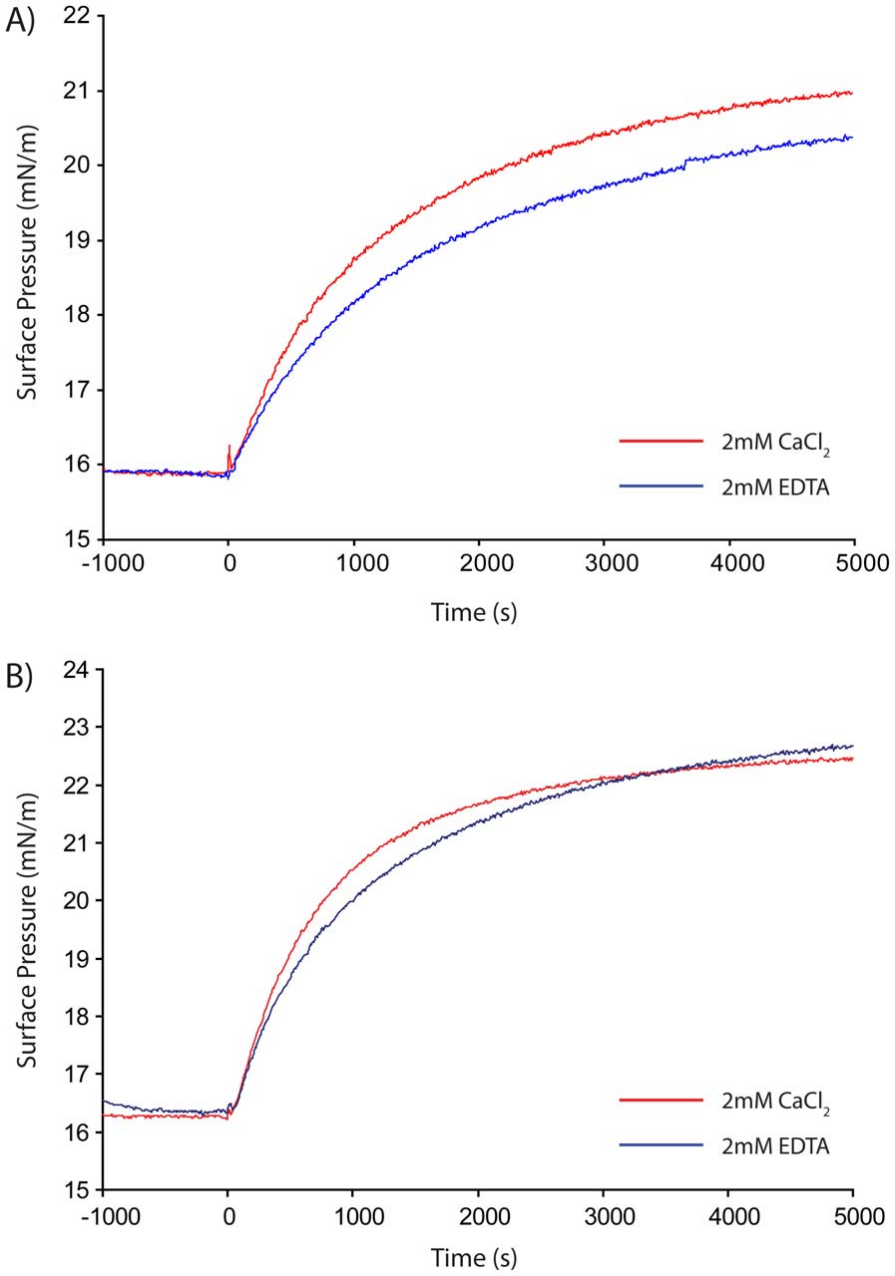
Adsorption of VILIP-1 to DOPS/DOPC (3∶1) phospholipid monolayers. Representative binding isotherms of myristoylated and unmyristoylated VILIP-1 are shown in panel A and B, respectively. In both panels, the red curve follows the adsorption of the protein in the presence of 2 mM CaCl_2_, while the blue curve follows the adsorption with 2 mM EDTA in the buffer.

Previous studies have shown that VILIP-1 acts at the membrane where it regulates the function of membrane receptors, e.g. GC [8], [36]. Since we were interested in the functional behaviour of myristoylated and unmyristoylated VILIP-1, we expressed and purified recombinant native and post-translationally modified form of the protein. Both unmyristoylated and myristoylated VILIP-1 were subjected to phospholipid monolayer adsorption experiments using a Langmuir surface film balance, and their membrane-binding activity was assessed in the presence and absence of calcium. As shown in Figure 2A, myristoylated VILIP-1 can bind lipid membranes in the presence and absence of calcium, with the extent of binding being marginally larger in the presence of calcium (see Table S1). This membrane-binding behaviour is similar to myristoylated frequenin/NCS-1, which has a constitutive membrane association independent of calcium binding [37]. Unmyristoylated VILIP-1 showed a membrane-binding behaviour (Figure 2B) similar to myristoylated VILIP-1, suggesting that both forms of VILIP-1 may have similar structural properties. In this paper, we consider the macromolecular structures of myristoylated and unmyristoylated VILIP-1.

### Quaternary structure in solution

**Figure 3:**
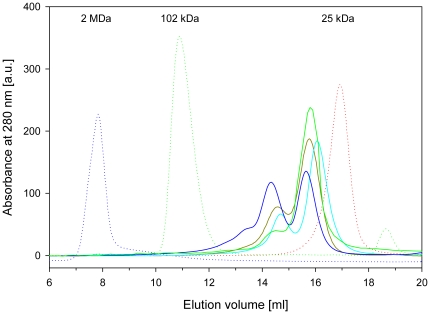
Size exclusion chromatograms of VILIP-1 samples with varying buffer conditions (solid lines). Non-reduced VILIP-1 with 0.1 mM EDTA (dark green), non-reduced VILIP-1 with 5 mM CaCl_2_ (blue), reduced VILIP-1 with 0.1 mM EDTA (light green), reduced VILIP-1 with 5 mM CaCl_2_ (cyan). Reduced samples were obtained by addition of 2.5 mM DTT to the buffer. The dotted lines show the chromatograms of selected protein standards; blue dextran (blue), SSB-301 (green) and chymotrypsinogen A (red).

Size exclusion chromatography of unmyristoylated VILIP-1 under varying conditions revealed two peaks that can be attributed to a monomeric and a dimeric species (see Figure 3), which have theoretical molecular masses of 22.1 and 44.8 kDa, respectively. Although the molecular mass of the dimer calculated from its elution time (i.e. 44.8 kDa) is in excellent agreement with its theoretical mass, the calculated molecular mass of the monomer (i.e. 33.6 kDa) is considerably larger than the theoretical mass. This suggests that the overall shape of the monomer is possibly prolate, causing it to elute faster than expected.

These results are in qualitative agreement with previous reports [19], [20], confirming that unmyristoylated VILIP-1 exists as a mixture of monomers and dimers in solution. Earlier studies have also shown that myristoylation does not affect the dimerisation behaviour of VILIP-1 [20]. From the current results, it appears that dimerisation is affected to some extent by divalent metal ions, but even more so by the addition of reducing agents, which decreases the relative amounts of dimers (see Table S2). Notably, a dimeric species still exists in reducing conditions, suggesting that formation of the dimer is not solely mediated by disulfide bonds. Therefore, the dimerisation of VILIP-1 is mediated by both Redox-dependent and -independent mechanisms.

### Small-angle X-ray scattering of reduced VILIP-1

In the absence of structural information at atomic resolution, we conducted small-angle X-ray scattering (SAXS) of the calcium-bound unmyristoylated VILIP-1 in solution. We chose the calcium-bound form because calcium significantly stabilises the tertiary structure of VILIP-1 [20]. The unmyristoylated form was used because calcium-bound myristolyated VILIP-1 is not very soluble and forms large protein aggregates in solution (at high protein concentrations) which is attributed to the calcium-induced exposure of the myristoyl group [20]. Under reducing conditions, where the formation of covalent dimeric species is suppressed, good quality SAXS data were acquired. Samples that were prepared under oxidising conditions did not yield usable data (not shown).

From the scattering curves, apparent values for radius of gyration and molecular masses were calculated (see Table 1). For the dataset at ρ* = 12 mg mL^−1^ the results are in agreement with an almost pure dimeric state, and at ρ* = 6 mg mL^−1^ with a monomeric state. For the dataset at ρ* = 3 mg mL^−1^, the calculated radius of gyration and molecular mass is much larger than at 6 mg mL^−1^. The distance distribution function reveals that the low concentration sample contains a large fraction of monomers (due to the good agreement between the *p*(*r*) curves at low *r* values) but also species with larger sizes as obvious from the long tail (*r*_max_∼260 Å; see Figure S1), indicating the presence of larger aggregates. This may be a result of radiation damage to the sample because larger aggregates were not observed at the other two concentrations.

#### Monomer

**Figure 4:**
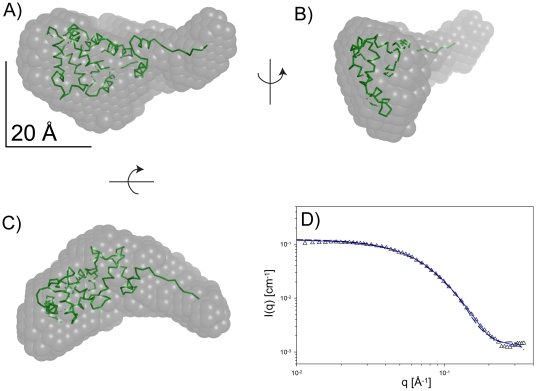
*Ab initio* shape restoration from the scattering data obtained with VILIP-1 in the presence of calcium (5 mM CaCl_2_) under reducing conditions (2.5 mM DTT) from the sample at ρ* = 6 mg/mL. Panels A–C show the superposition of the protein model of calcium-bound VILIP-1 with the restored shape in three orthogonal views. Panel D shows the comparison of the theoretical and experimental scattering. Theoretical scattering data from the GASBOR [30] shape (dashed black line) and the atomic model (solid blue line) are shown.

The dataset at ρ* = 6 mg mL^−1^ (buffer conditions: 100 mM NaCl, 5 mM CaCl_2_, 2.5 mM DTT, 20 mM HEPES, pH 7.5) allows conclusions as to the structure of monomeric VILIP-1, within the experimental error. The experimentally determined molecular mass of 25.9 kDa is very close to the theoretical value (22.1 kDa). Using GASBOR [31], an *ab initio* model of the shape of monomeric VILIP-1 was generated assuming no symmetry (see Figure 4). The restored shape is reminiscent of the open, calcium-bound structure, which agrees with the presence of 5 mM Ca^2+^ in the sample.

#### Dimer under reducing conditions

**Figure 5:**
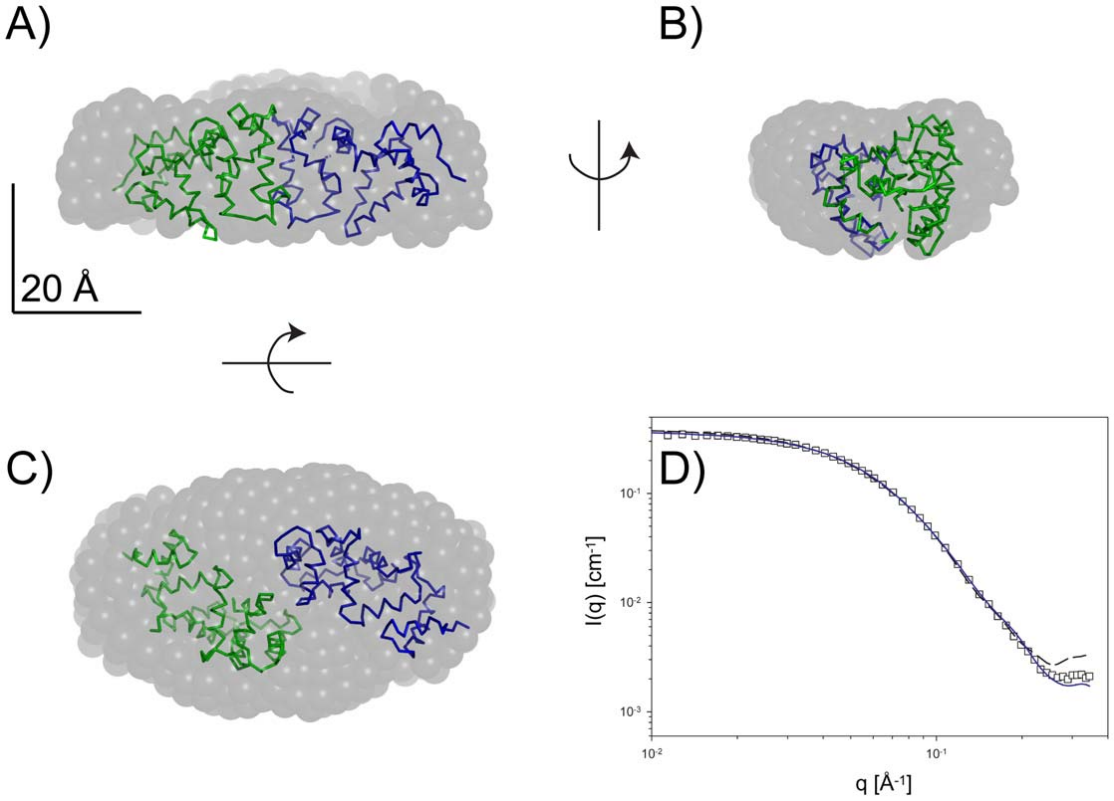
Low-resolution structure of the VILIP-1 dimer under reducing conditions. Shown in three orthogonal views is the restored shape obtained from the sample at ρ* = 12 mg/mL (Panels A–C) superimposed over the calcium-bound VILIP-1 dimer model. Panel D shows the comparison of the theoretical and experimental scattering. Theoretical scattering data from the GASBOR [30] shape (dashed black line) and the atomic model (solid blue line) are shown.

The data collected for the sample of reduced VILIP-1 at 12 mg mL^−1^ (buffer conditions: 100 mM NaCl, 5 mM CaCl_2_, 2.5 mM DTT, 20 mM HEPES, pH 7.5) indicates the presence of mainly dimeric VILIP-1. The experimentally determined molecular mass from the intensity of forward scattering (42 kDa) is very close to the expected dimer mass, suggesting that this dataset is well suited for modelling dimeric VILIP-1 in solution. *Ab initio* shape restoration with GASBOR clearly yielded a prolate envelope large enough to accommodate two VILIP-1 monomers in their open conformation (see Figure 5).

### Model construction

#### Monomer

We attempted to fit the dataset at ρ* = 6 mg mL^−1^ with our homology model of calcium-bound VILIP-1 as a rigid body, but this left some portion of the SAXS-derived shape unaccounted for. The initial fit between theoretical and experimental scattering data as calculated with CRYSOL [31] was moderate with χ = 8.85. At the current state of knowledge, the very N-terminal residues of VILIP-1 are not involved in packing interactions of the overall protein fold, and therefore can be assumed to be rather flexible. Accordingly, when using residue Pro9 as a hinge between the very N-terminal region and helix α1, models can be generated that show an improved fit to the SAXS-derived shape of monomeric VILIP-1 (see Figure 4). These monomeric VILIP-1 models have the very N-terminal region pointing away from the core of the molecule, with some degree of flexibility. A representative conformation is shown in Figure 4. In addition, the sample at ρ* = 6 mg mL^−1^ contains a small fraction of dimers. Adding a constant background term to account for flexibility, as well as a small fraction of dimers, the fit between theoretical and experimental scattering data could be improved to χ = 1.87. The radius of gyration increased from *R_g_* = 21.8 Å (initial model) to *R_g_* = 22.1 Å (final model), bringing it marginally closer to the experimental value of *R_g_* = 25.9 Å.

#### Dimer under reducing conditions

In previous mutation studies, specific residues located in EF3 and EF4 of VILIP-1 have been identified to be important for the formation of the dimer. By applying these experimental observations as restraints in protein docking a model of the non-covalent VILIP-1 dimer has been proposed [20]. In the current study, we fitted the proposed dimer into the low resolution shape derived from the SAXS data as a starting conformation with only minor manual adjustment. With subsequent computational rigid-body refinement against the scattering data (ρ* = 12 mg mL^−1^) a final model was obtained with a goodness of fit of χ = 2.5.

Structural information for another VILIP dimer conformation is available for bovine neurocalcin δ, for which a dimer in the crystal structure (PDB accession code 1BJF) has been reported [22]. However, the dimer interface in this case is generated by three EF-hand loops (EF2-EF4), resulting in a rather globular shape, as opposed to the prolate shape found experimentally with VILIP-1. The crystal structure of neurocalcin δ was thus not considered as a template for modelling.

## Discussion

A number of NCS proteins (e.g. DREAM [23], neurocalcin δ [22], and GCAP2 [12]) undergo functional dimerization. In this study, we confirm that VILIP-1 exists in monomeric and dimeric forms, and propose models of their structures based on solution scattering data. Combining these structural models with results from monolayer binding experiments, we propose a mechanism of action for VILIP-1 in the cellular environment.

**Figure 6:**
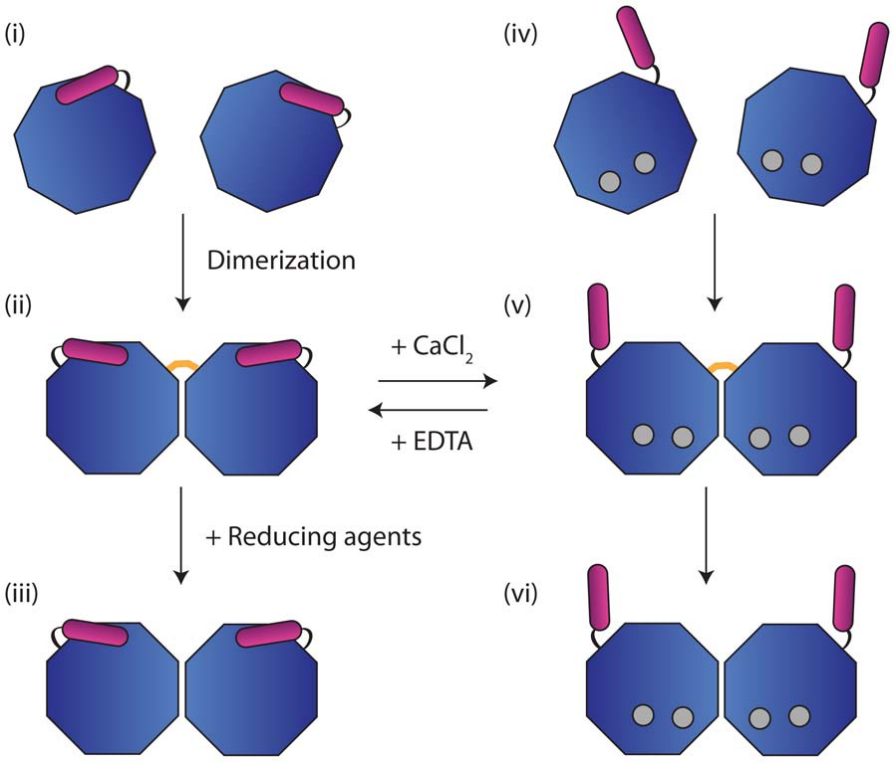
Different structural species of VILIP-1 in solution. The species include: (i) Monomer of the protein in the absence of calcium; (ii) Disulphide-linked dimer of the protein in the absence of calcium; (iii) Non-covalent dimer of the protein in the absence of calcium; (iv) Monomer of the calcium-bound protein; (v) Disulphide-linked dimer of the calcium-bound protein; and (vi) Non-covalent dimer of the calcium-bound protein. The core domain of VILIP-1 is coloured blue, the myristoyl group is coloured purple, and bound calcium ions are coloured grey.

The present results from size exclusion chromatography reveal that divalent cations and Redox conditions induce dimer formation. In solution, VILIP-1 exists as a mixture of different species (see Figure 6):

- Monomer of the protein in the absence of calcium
- Disulphide-linked dimer of the protein in the absence of calcium
- Non-covalent dimer of the protein in the absence of calcium
- Monomer of the calcium-bound protein
- Disulphide-linked dimer of the calcium-bound protein
- Non-covalent dimer of the calcium-bound protein

Indeed, the existence of a complex mixture of VILIP-1 species may help explain why NMR spectra acquired for VILIP-1 display significant amounts of spectral heterogeneity, preventing determination of its solution structure [20]. Using small-angle X-ray scattering as a complementary technique to investigate the low resolution solution structure, we have obtained structural information for monomers and dimers of unmyristoylated VILIP-1.

For the monomeric species (sample concentration of 6 mg mL^−1^), the overall shape restored from the SAXS data is prolate. This deviation from an idealised globular shape agrees with the apparent molecular mass obtained from size exclusion chromatography, which was larger than the theoretical mass. A homology model of monomeric calcium-bound VILIP-1 fitted well into the SAXS-derived shape, showing that the N-terminal region (residues 1–9) is exposed and rather flexible. NMR-based experiments on myristoylated VILIP-1 confirm that the N-terminal region is exposed in the calcium-bound state [20].

Comparison of our homology models of VILIP-1 in the calcium-free (i) and calcium-bound states (iv) shows that there are structural rearrangements in the orientation of EF1 and EF2, but EF3 and EF4 remain relatively fixed and rigid. The structural differences of VILIP-1 that can be induced by calcium is functionally relevant, as the concentration of calcium is believed to regulate the biological function of VILIP-1 in the cellular environment.

The non-covalent calcium-bound VILIP-1 dimer observed in the current SAXS study (sample concentration 12 mg mL^−1^) is constituted by an interface formed by EF3 and EF4. Since this interface is independent of and most probably unaffected by calcium binding, the packing of the monomers in the calcium-free (iii) and calcium-bound (vi) dimers should be similar. Furthermore, calcium binding is expected to induce extrusion of the N-terminal myristoyl groups, which are located away from the dimer interface. Notably, the overall shape is distinctly different to the dimer models of other NCS proteins, such as recoverin [21], DREAM [23], and neurocalcin δ [22], suggesting that although NCS proteins share a similar overall fold, they may have different modes of action.

It is unclear whether the disulphide-linked dimers in the absence (ii) and presence of calcium (v) are similar to the non-convalent dimers as discussed above. Chen and coworkers reported that a Cys187Ala mutant of VILIP-1 showed less dimer formation and significantly reduced GC activation [19], suggesting that dimer formation is functionally significant and Cys187, located at the flexible C-terminal end, is responsible for covalent dimer formation. They also showed that formation of the disulphide-linked dimer is reversible [19]. It is unclear whether the other two cysteine residues (Cys38 and Cys87) of VILIP-1 are involved in dimer formation. It seems unlikely that an intra-molecular disulfide bond is involved in dimer formation because of the large inter-atomic distances separating each Cys-Cys residue pair (see Table S3).

**Figure 7:**
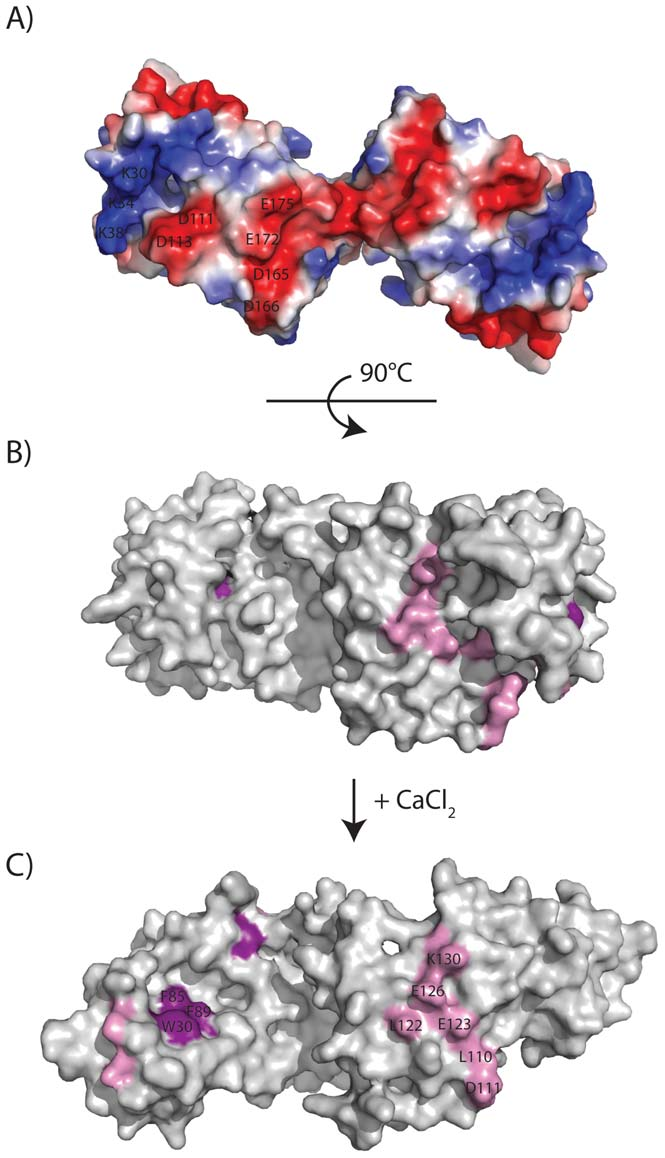
Potential interaction surfaces of the VILIP-1 dimer. Panel A shows the electrostatic surface potential of the calcium-free VILIP-1 dimer. This highly charged surface may be important for membrane binding. Panel B and C show predicted interaction surfaces for guanylyl cyclases (pink) and nicotinic acetylcholine receptors (purple). Panel B shows the closed conformation with the myristoyl group sequestered, and panel C shows the open conformation with myristoyl group exposed upon VILIP-1 binding calcium.

For recoverin, the prototypical NCS protein, the classical calcium-myristoyl switch regulates its binding to lipid membranes. As it has been recently shown that the myristoyl group of VILIP-1 becomes exposed upon calcium binding [20], we employed a model membrane system to study the interaction of VILIP-1 with lipid membranes. We were surprised to find that both unmyristoylated and myristoylated VILIP-1 can bind lipid membranes, and binding is largely independent of calcium, which agrees with previous Western blot experiments on VILIP-1 [38]. This suggests that other residues of VILIP-1 are important for membrane binding; indeed a similar observation has been observed for GCAP-2, where its interaction with lipid membranes is mainly driven by protein side chain-lipid interactions [39]. Analysis of the dimer model shows that it has a highly charged region on its surface composed of residues from EF1 (e.g. Lys30, Lys34, Lys38), EF3 (e.g. Asp111, Asp113), and EF4 (e.g. Asp165, Asp166, Glu172, Glu175), which may facilitate interaction with charged membranes through charge-charge interactions (see Figure 7A). Future studies may study the dissociation behaviour of VILIP-1 to better understand the structural features that modulate its interaction with lipid membranes. Since the myristoyl group is not required for membrane binding, does the calcium-myristoyl switch in VILIP-1 serve a different purpose? One possibility may be a role in the activation of a binding partner, as proposed for other calcium-myristoyl-switch proteins by Meyer and York [40].

Guanylyl cyclase has been proposed as a potential binding partner for VILIP-1, its surface expression and activity can be regulated by VILIP-1 [9]. Based on experimental data on the interaction between GCAP1 and retinal GC [16], we have previously mapped residues on our VILIP-1 homology model that are putatively important for interactions with guanylyl cyclase [32]. These interactions with the membrane receptor can occur in the interfacial groove area between EF3 and EF4, which is distinct from the dimerization surface, as shown in Figure 7B.

VILIP-1 has also been shown to facilitate the calcium-induced trafficking of the alpha4-subunit of nicotinic acetylcholine receptors [41]. It has been proposed that an exposed hydrophobic crevice on the surface of the calcium-bound dimer serves as a target binding site, similar to that observed previously in the structures of recoverin bound to rhodopsin kinase [42] and yeast frequenin bound to PtdIns 4-kinase [43].

A shared feature of all proposed target protein binding sites is that they are only fully exposed when the NCS protein is bound to calcium (Figure 7B). In the calcium-free VILIP-1 dimer, the interaction sites are either partially or fully occluded, preventing binding to a target protein (Figure 7C). In support of this theory, surface plasmon resonance experiments have shown that the binding of VILIP-1 to guanylyl cyclase requires calcium [9].

**Figure 8:**
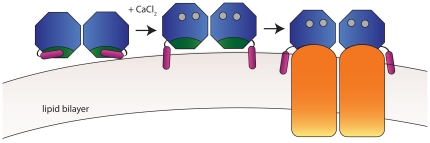
Molecular mechanism of VILIP-1. In the resting state of the cell, VILIP-1 has the ability to bind membranes. When the intracellular calcium concentration increases, the myristoyl group (coloured purple) is extruded. This exposes the target binding site (coloured green), allowing the VILIP-1 dimer to interact with the target protein (coloured orange).

Based on our analysis, we hypothesize a molecular mechanism for VILIP-1, as shown in Figure 8. In the neuronal resting state, the calcium-free VILIP-1 dimer sits at the periphery of membranes (e.g. of the ER) inside the cell in a “closed” conformation. Indeed, at resting calcium levels in cerebellar granule cells, VILIP-1 is associated with the plasma membrane and distributed throughout the cytosol [38]. Upon stimulation and increase of cytosolic calcium levels, VILIP-1 binds calcium, leading to extrusion of the myristoyl group and formation of the “open” conformation, exposing the target protein interaction site to its binding partner at the membrane. Thus, we propose that VILIP-1 helps to activate proteins at the membrane in response to calcium signals.

Direct interactions between VILIP-1 and guanylyl cyclase have been observed [11], and physiological regulation of the guanylyl cyclase is hypothesised to require dimerisation of VILIP-1. Based on the low-resolution solution structure of dimeric VILIP-1, this study provides a structural model and testable hypotheses for VILIP-1 interactions with guanylyl cyclase. Studies to clarify the molecular and structural details of VILIP-1 interactions with its receptor are currently under way in our laboratories.

## Supporting Information

Figure S1 **Small-angle X-ray scattering data obtained from VILIP-1 under reducing conditions (2.5 mM DTT) in the presence of calcium (5 mM CaCl_2_).** ***Left***: Plot of the scattering intensity against the scattering vector *q* and fit of the data obtained: ρ* = 12 mg mL^−1^ (squares), ρ* = 6 mg mL^−1^ (triangles), ρ* = 3 mg mL^−1^ (circles). The solid lines are the theoretical fits obtained by the IFT approach. ***Right***: Pair distance distribution functions *p(r)* calculated by the IFT method. Results for all three measured samples are shown: ρ* = 12 mg mL^−1^ (solid line), ρ* = 6 mg/mL (dashed line), ρ* = 3 mg/L ^−1^(dotted line). The *p*(*r*) curves were normalised by concentration. The distance distribution function of the sample at ρ* = 3 mg mL^−1^ shows the presence of large particles (r_max_∼260 Å).
(PDF)

Table S1 **Statistical analysis of monolayer experiments.**
(PDF)

Table S2 **Quaternary structure from size exclusion chromatography.**
(PDF)

Table S3 **Inter-atomic Cα- Cα distances between Cys residue pairs of VILIP-1.**
(PDF)
