# Supplementary figures and images for: Divalent Cations and Redox Conditions Regulate the Molecular Structure and Function of Visinin-Like Protein-1
Source: PLoS One. 2011 Nov 2;6(11):e26793. doi: 10.1371/journal.pone.0026793 (PMC3206844; doi:10.1371/journal.pone.0026793)

Supporting Figure S1

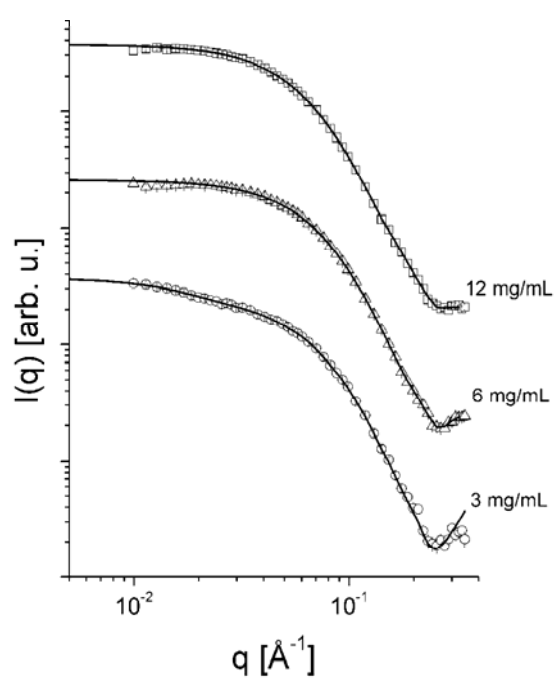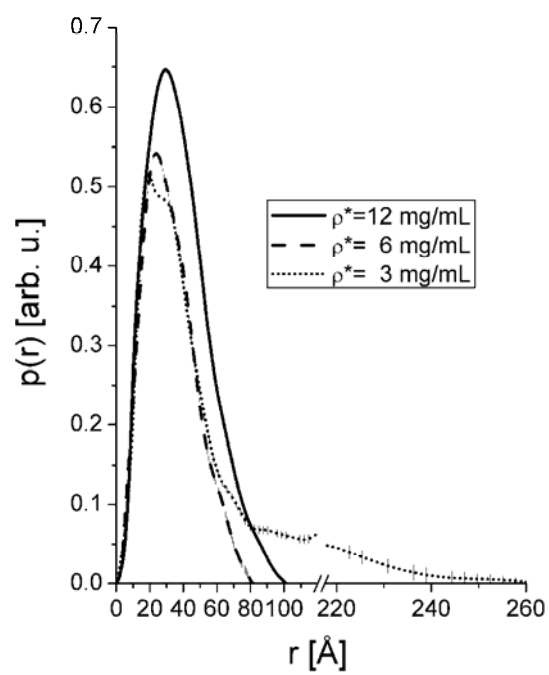

Supplement: Figure S1 — Small-angle X-ray scattering data obtained from VILIP-1 under reducing conditions (2.5 mM DTT) in the presence of calcium (5 mM CaCl2). Left: Plot of the scattering intensity against the scattering vector q and fit of the data obtained: ρ* = 12 mg mL−1 (squares), ρ* = 6 mg mL−1 (triangles), ρ* = 3 mg mL−1 (circles). The solid lines are the theoretical fits obtained by the IFT approach. Right: Pair distance distribution functions p(r) calculated by the IFT method. Results for all three measured samples are shown: ρ* = 12 mg mL−1 (solid line), ρ* = 6 mg/mL (dashed line), ρ* = 3 mg/L −1(dotted line). The p(r) curves were normalised by concentration. The distance distribution function of the sample at ρ* = 3 mg mL−1 shows the presence of large particles (rmax∼260 Å). (PDF) [file pone.0026793.s001.pdf]
