# Supplementary material for: Divalent Cations and Redox Conditions Regulate the Molecular Structure and Function of Visinin-Like Protein-1
Source: PLoS One. 2011 Nov 2;6(11):e26793. doi: 10.1371/journal.pone.0026793 (PMC3206844; doi:10.1371/journal.pone.0026793)
Supplement: Table S1 — Statistical analysis of monolayer experiments. (PDF) [file pone.0026793.s002.pdf]

Supporting Table S1: Statistical analysis of monolayer experiments

	**$CaCl_2$**	**EDTA**
**Myr-VILIP-1**	5.4 ± 0.2 mN/m	4.6 ± 0.5 mN/m
**Unmyr-VILIP-1**	5.9 ± 0.1 mN/m	6.0 ± 0.6 mN/m

*Mean surface pressure values obtained after the adsorption of proteins to lipid monolayers in 20 mM HEPES at pH 7.5, 100 mM NaCl and 2 mM $CaCl_2$ or 2 mM EDTA. The values are averages of at least three binding isotherms.
