# Supplementary material for: Divalent Cations and Redox Conditions Regulate the Molecular Structure and Function of Visinin-Like Protein-1
Source: PLoS One. 2011 Nov 2;6(11):e26793. doi: 10.1371/journal.pone.0026793 (PMC3206844; doi:10.1371/journal.pone.0026793)
Supplement: Table S2 — Quaternary structure from size exclusion chromatography. (PDF) [file pone.0026793.s003.pdf]

Supporting Table S2: Quaternary structure from size exclusion chromatography

**Protein**	**DTT**	**$CaCl_2$**	**EDTA**	***M* (monomer) [kDa]**	***M* (dimer) [kDa]**	***x* (monomer) %**	***x* (dimer) %**
VILIP-1	2.5 mM	-	0.1 mM	32.9	45.4	84	16
VILIP-1	2.5 mM	5 mM	-	31.7	44.2	73	27
VILIP-1	-	-	0.1 mM	34.1	45.2	68	32
VILIP-1	-	5 mM	-	34.2	47.1	42	58
