# Supplementary material for: Divalent Cations and Redox Conditions Regulate the Molecular Structure and Function of Visinin-Like Protein-1
Source: PLoS One. 2011 Nov 2;6(11):e26793. doi: 10.1371/journal.pone.0026793 (PMC3206844; doi:10.1371/journal.pone.0026793)
Supplement: Table S3 — Inter-atomic Cα- Cα distances between Cys residue pairs of VILIP-1. (PDF) [file pone.0026793.s004.pdf]

Supporting Table S3: Inter-atomic Cα- Cα distances between Cys residue pairs of VILIP-1

	**Cys38**	**Cys87**	**Cys187**
**Cys38**	**-**	17.34 Å	27.73 Å
**Cys87**	17.34 Å	-	11.03 Å
**Cys187**	27.73 Å	11.03 Å	-

*Distances were calculated using PyMOL and are based on the calcium-bound VILIP-1 dimer in reducing conditions.
